# Kinetics of Molybdenum Reduction to Molybdenum Blue by *Bacillus* sp. Strain A.rzi

**DOI:** 10.1155/2013/371058

**Published:** 2013-12-02

**Authors:** A. R. Othman, N. A. Bakar, M. I. E. Halmi, W. L. W. Johari, S. A. Ahmad, H. Jirangon, M. A. Syed, M. Y. Shukor

**Affiliations:** ^1^Department of Biochemistry, Faculty of Biotechnology and Biomolecular Sciences, University Putra Malaysia, 43400 Serdang, Selangor, Malaysia; ^2^Department of Environmental Science, Faculty of Environmental Studies, Universiti Putra Malaysia, 43400 Serdang, Selangor, Malaysia; ^3^Department of Microbiology, Faculty of Biotechnology and Biomolecular Sciences, University Putra Malaysia, 43400 Serdang, Selangor, Malaysia

## Abstract

Molybdenum is very toxic to agricultural animals. Mo-reducing bacterium can be used to immobilize soluble molybdenum to insoluble forms, reducing its toxicity in the process. In this work the isolation of a novel molybdate-reducing Gram positive bacterium tentatively identified as *Bacillus* sp. strain A.rzi from a metal-contaminated soil is reported. The cellular reduction of molybdate to molybdenum blue occurred optimally at 4 mM phosphate, using 1% (w/v) glucose, 50 mM molybdate, between 28 and 30°C and at pH 7.3. The spectrum of the Mo-blue product showed a maximum peak at 865 nm and a shoulder at 700 nm. Inhibitors of bacterial electron transport system (ETS) such as rotenone, sodium azide, antimycin A, and potassium cyanide could not inhibit the molybdenum-reducing activity. At 0.1 mM, mercury, copper, cadmium, arsenic, lead, chromium, cobalt, and zinc showed strong inhibition on molybdate reduction by crude enzyme. The best model that fitted the experimental data well was Luong followed by Haldane and Monod. The calculated value for Luong's constants *p*_max_, *K*_*s*_, *S*_*m*_, and *n* was 5.88 **μ**mole Mo-blue hr^−1^, 70.36 mM, 108.22 mM, and 0.74, respectively. The characteristics of this bacterium make it an ideal tool for bioremediation of molybdenum pollution.

## 1. Introduction

Heavy metals are ubiquitously applied in numerous industrial processes. This has resulted in heavy metals contamination of many environmental systems in Malaysia [1–3]. Molybdenum is an example of a heavy metal with numerous applications in industries. It is a significant pollutant with levels as high as thousands of ppm found in aquatic bodies and soils [4, 5]. The toxicity of molybdenum compounds has been studied intensively in animals [6, 7]. Cows are the most affected with dramatic scouring occurring at 20–50 mg Mo/kg body weight, followed by sheep and pigs [8]. In Malaysia, molybdenum is produced as a byproduct of copper from a mine in Sabah [9]. 

Like other heavy metals pollution, scientists have turned towards bioremediation, a cheaper alternative using the ability of microbe to remove and resist heavy metals via mechanisms such as sequestration, bioreduction, biosorption, transport mechanisms, bioprecipitation, and/or chelation [10]. Microbial reduction of molybdate (Mo^6+^) to Mo-blue was first reported in 1896 [11]. Since the last thirteen years, almost all of the reported bacteria capable of microbiological reduction of molybdate to molybdenum blue [12–25] came from our work. Screening for novel molybdenum reducers is required in order to develop a cost-effective bioremediation work for cleaning up molybdenum pollutants in the environment [26]. Numerous commercial bioremediating bacteria use *Bacillus* spp. as this genus offers several advantage such as extreme environmental tolerance due to its capability to form endospore and a fast doubling time [27]. In this work, we report on the isolation of a novel molybdenum-reducing *Bacillus *sp. strain A.rzi and its kinetics of molybdenum reduction to molybdenum blue (Mo-blue).

## 2. Experimental

### 2.1. Molybdenum-Reducing Bacterium Isolation

Soil samples were taken from a metal recycling plant in Kajang, Selangor, in December 2007. Soil sample (five gram) was suspended in 50 mL of phosphate buffered saline (1x). Suitable serial dilutions (aliquot 0.1 mL) were spread onto molybdenum selective agar of low phosphate (2.9 mM phosphate) media (pH 7.5) supplemented with glucose (10 gL^−1^) as the carbon source, NaCl (5 gL^−1^), (NH_4_)_2_SO_4_ (3 gL^−1^), Na_2_MoO_4_·2H_2_O (2.42 gL^−1^), MgSO_4_·7H_2_O (0.5 gL^−1^), yeast extract (0.5 gL^−1^), and Na_2_HPO_4_·2H_2_O (0.51 gL^−1^) [17]. The strongest blue colony on the plate was transferred into 50 mL of liquid low phosphate media. Molybdenum-reducing bacterial strains such as *Serratia *sp. strain Dr.Y8, *Serratia *sp. strain Dr.Y5,* S. marcescens *strain Dr.Y9, *Enterobacter *sp. strain Dr.Y13, *Pseudomonas *sp. strain DRY2, *Serratia marcescens *strain DRY6, *Acinetobacter calcoaceticus *strain Dr.Y12, and *Enterobacter cloacae *strain 48 were obtained from our culture collection and *E. coli* K12 was obtained from American Type Culture Collection, Rockville, USA. The bacteria were grown and maintained on the above low phosphate liquid and solid media, respectively. 

### 2.2. Molyvbdenum-Reducing Bacterium Identification

Identification of the bacterium was performed by using Biolog GP microplate (Biolog, Hayward, CA, USA) according to the manufacturer's instructions and molecular phylogenetic studies. Genomic DNA was prepared through alkaline lysis method. PCR amplification was carried out using a Biometra T Gradient PCR (Montreal Biotech Inc., Kirkland, QC). The PCR mixture comprises 0.5 pM of the following primers: 5′-AGAGTTTGATCCTGGCTCAG-3′ and 5′-AAGGAGGTGATCCAGCCGCA-3′ corresponding to the forward and reverse primers of 16S rDNA, respectively [28], 2.5 U of Taq DNA polymerase (Promega), 200 *μ*M of each deoxynucleotide triphosphate, and 1x reaction buffer. The reaction mixture had a final volume of 50 *μ*L. The 16S rDNA gene from the genomic DNA was amplified by PCR using the following conditions: an initial denaturation at 94°C for 3 min; 25 cycles at 94°C for 1 min, 50°C for 1 min, and 72°C for 2 min; and a final extension at 72°C for 10 min. The Big Dye terminator kit (Perkin-Elmer Applied Biosystems) was used for cycle sequencing. The resultant 1306 bases were compared to similar sequence in the GenBank database using the NCBI Blast server (http://blast.ncbi.nlm.nih.gov/Blast.cgi). The 16s rRNA ribosomal gene sequence was deposited in GenBank under the accession number EU835195.

### 2.3. Phylogenetic Analysis

Alignment of 20 16S rRNA gene sequences closely matching strain A.rzi retrieved from GenBank was carried out using clustal_W [29]. The construction of the phylogenetic tree was carried out using PHYLIP, version 3.573 (J. Q. Felsenstein, PHYLIP—phylogeny inference package, version 3.573, Department of Genetics, University of Washington, Seattle, WA (http://evolution.genetics.washington.edu/phylip.html)). *S. marcescens* was the outgroup in the cladogram. The neighbour-joining/UPGMA method was used to construct the evolutionary distance matrices the DNADIST algorithm program. A distance matrix was used instead of the laborious maximum likelihood or parsimony approaches. The model used in the nucleotide substitution is from Jukes and Cantor [30]. Phylogenetic tree was constructed based on the neighbour-joining method adopted from Saitou and Nei [31]. Confidence levels for individual branches within the tree were checked for each algorithm by repeating the PHYLIP analysis with 1000 bootstraps [32] using the SEQBOOT program in the PHYLIP package. The jackknife approach can also be used. Majority rule (50%) consensus trees were constructed using the Ml methods [33]. The tree was viewed using the TreeView program [34].

### 2.4. Crude Enzyme Preparation

The preparation of crude enzyme was based on the modified method of Shukor et al. [35]. Experiments were carried out at 4°C unless stated otherwise. A 2 L culture was grown overnight on high phosphate media (HPM) containing MgSO_4_·7H_2_O (0.5 gL^−1^), NaCl (5 gL^−1^), (NH_4_)_2_SO_4_ (3 gL^−1^), NaMoO_4_·2H_2_O (12.1 gL^−1^ or 50 mM), yeast extract (1 gL^−1^), glucose (10 gL^−1^) as an electron donor source, and Na_2_HPO_4_ (100 mM) at pH 7.3. The bacterial cells were first harvested at 10000 ×g for 20 min at 4°C. The pellet was washed several times and reconstituted with 10 mL of 50 mM Tris buffer (pH 7.0) containing 1 mM phenylmethanesulphonyl sfluoride (PMSF) as a protease inhibitor and 2 mM of DTT as a reducing agent for protecting the thiol group in the enzyme. The cells were then subjected to sonication on a Biosonik 111 sonicator for a total sonication time of 2 hours with intermittent cooling on an ice bath and then ultracentrifuged at 105000 ×g for 90 min at 4°C. The supernatant is the crude enzyme and was used for further studies. Enzyme was assayed according to the method of Shukor et al. [35] by adding 100 *μ*L of NADH (80 mM stock) 1 mL of a reaction mixture consisting of laboratory-prepared phosphomolybdate (LPP) electron acceptor substrates prepared in 50 mM citrate-phosphate buffer pH 5.0 at room temperature. The final concentration of LPP was 8 mM. To start the reaction, fifty microlitres of crude Mo-reducing enzyme were added. The absorbance increase in a one minute incubation period was read at 865 nm. The definition of one unit of Mo-reducing activity is the amount of enzyme that produces 1 nmole of Mo-blue per minute at room temperature. The specific extinction coefficient for the product Mo-blue at 865 nm is 16.7 mM^−1^·cm^−1^ [36]. 

### 2.5. Studies on the Effects of Metal Ions and Respiratory Inhibitors

Inhibitors such as sodium azide, antimycin A, rotenone, and potassium cyanide were dissolved in deionised water and/or in acetone [37]. Metal ions were dissolved in 20 mM Tris·Cl buffer (pH 7.0). A preincubation of the inhibitors or metal ions with one hundred microlitres of enzyme in the reaction mixture was carried out at 4°C for 10 minutes. The incubation mixture was warmed to room temperature before NADH was added to start the reaction. The total reaction mixture was 1.0 mL. As a control for inhibitors that was dissolved in acetone such as rotenone and antimycin A, 50 *μ*L of acetone was added in the reaction mixture without inhibitors. The linear increase in absorbance at 865 nm was measured after an incubation period of 5 minutes.

### 2.6. Characterization of the Molybdate Reduction Reaction Using the Dialysis Tubing Method

The dialysis tubing method is a modification of the method applied by Hem [41] to identify whether the reduction of heavy metals seen physiologically was due to biotic chemicals produced by the cells or catalyzed through an enzymatic route. The dialysis tubing method of Shukor et al. [23] was used in this work. 

### 2.7. Determination of Kinetic Parameters for Molybdate Reduction to Molybdenum Blue

Determination of intrinsic growth kinetic parameters for strain A.rzi was not possible due to the property of the molybdenum blue that form a precipitate together with the bacterial mass [18–25]. Hence, only the reduction kinetics was studied. Several substrate inhibition kinetic models available such as Haldane and Luong were compared to the commonly used Monod. In this work molybdenum reduction kinetics is represented as Mo-blue production rate. The formula for the above model is shown in Table 1, where *p*, *p*_max⁡_, *K*_*s*_, *K*_*i*_, *S*, *S*_*m*_, and *n* are specific Mo-blue production rate (hr^−1^), maximum Mo-blue production rate (hr^−1^), half-saturation constant (mM), inhibition constant (mM), substrate concentration (mM), critical substrate concentration above which production of Mo-blue completely stops (mM), and the exponent representing the impact of the substrate to *p*_max⁡_, respectively. The Mo-blue production rate is calculated based on the linear portion of the Mo-blue production against time.

### 2.8. Statistical Analysis

Comparison between groups was performed using a Student's *t*-test or a one-way analysis of variance (ANOVA) with post hoc analysis by Tukey's test. *P* < 0.05 was considered statistically significant.

## 3. Results

### 3.1. Identification of Mo-Reducing Bacterium

**Figure 1:**
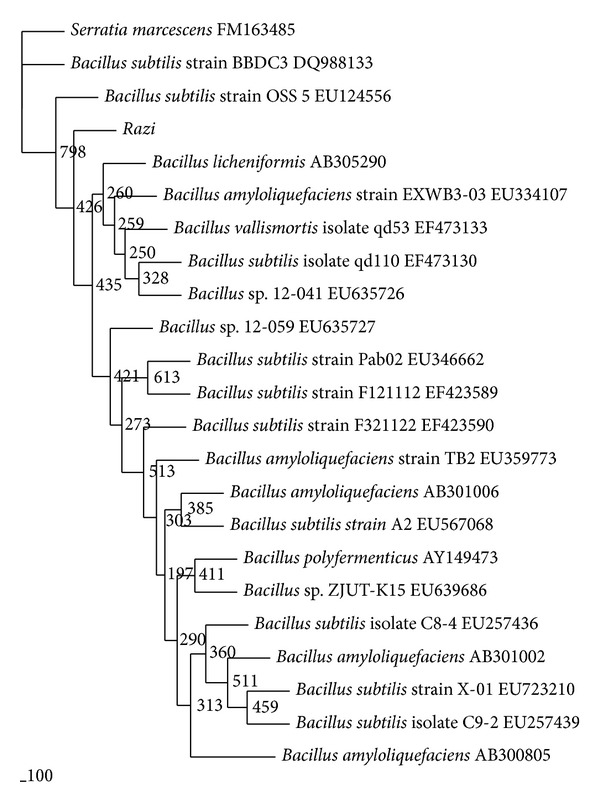
Phylogram (neighbour-joining method) indicating the 16s rRNA genetic relationship between 20 other related references microorganisms from the GenBank database and strain A.rzi. *S. marcescens* is the outgroup. Species names of bacteria were followed by the accession numbers of 16s rRNA. The internal labels at the branching points are the bootstrap value. *Scale bar* represents 100 nucleotides substitution.

A Gram-positive spore-forming bacterium capable of molybdenum reduction to molybdenum blue was isolated from a metal-contaminated soil. The bacterium was identified through phylogenetic analyses of the 16S rRNA ribosomal gene sequence of the bacterium. A high bootstrap value (>75%) of 79.8% was obtained when strain A.rzi is genetically linked to *Bacillus *sp. (Figure 1). This is a novel molybdenum-reducing Gram positive bacterium (Figure 1). The identifications performed using GP2 plate with the BIOLOG system gave 98% probability, 0.747 similarity, and 3.56 distance value to *Bacillus pumilus* B. At this juncture, we tentatively assigned this bacterium as *Bacillus* sp. strain A.rzi.

### 3.2. Comparison of Mo-Blue Production among Mo-Reducing Isolates

**Figure 2:**
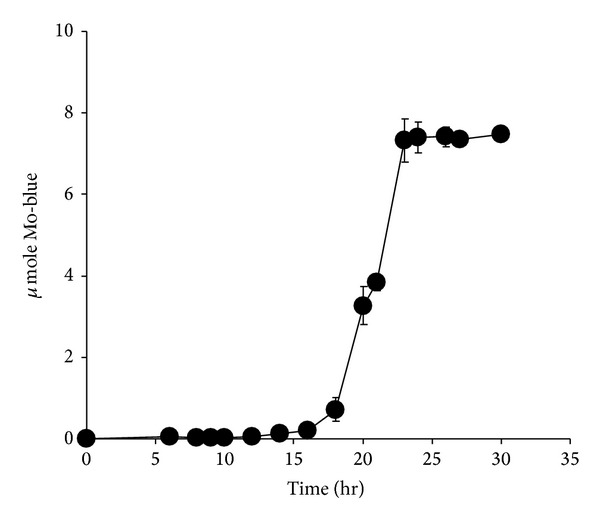
Time course profile of Mo-blue production from strain A.rzi.

When grown in 10 mM molybdate as the benchmark molybdate concentration, strain A.rzi ranked as the second best together with *Serratia *sp. strain Dr.Y5 in producing Mo-blue (Table 2). The optimal carbon sources of either sucrose or glucose supporting molybdate reduction for each bacterium [18–25] were used in this comparison work. Mo-blue production increases dramatically after 18 hours of static growth, reaching maximal production after 23 hours of incubation (Figure 2). 

### 3.3. The Effects of Phosphate and Molybdate Concentrations

**Figure 3:**
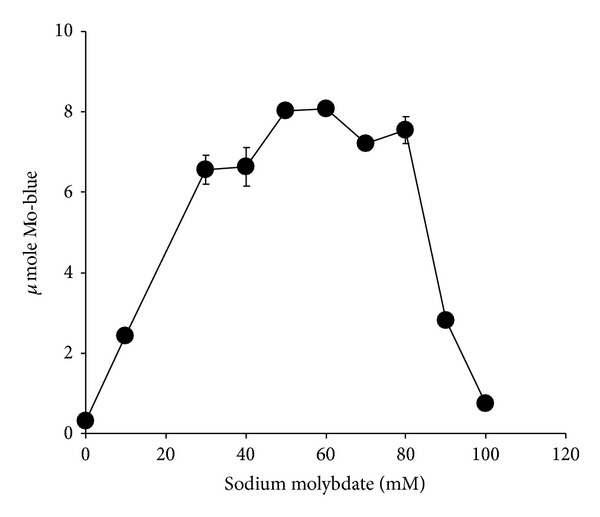
The effect of molybdate on molybdate reduction by strain A.rzi. Error bars represent mean ± standard error (*n* = 3).

**Figure 4:**
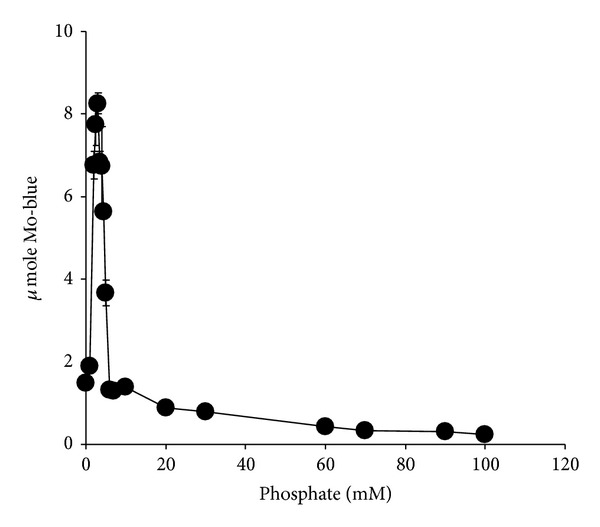
The effect of phosphate on molybdate reduction by strain A.rzi. Error bars represent mean ± standard error (*n* = 3).

The effect of molybdate concentration on molybdate reduction (Figure 3) showed that the optimum concentration of molybdate was between 50 and 60 mM. Concentrations higher than 80 mM molybdate were strongly inhibitory. At 50 mM molybdate, molybdate reduction was strongly critical of phosphate concentration with a sharp optimum at 4 mM and a near complete inhibition of Mo-reduction at higher phosphate concentrations (Figure 4). 

### 3.4. The Effect of pH and Temperature on Molybdate Reduction

**Figure 5:**
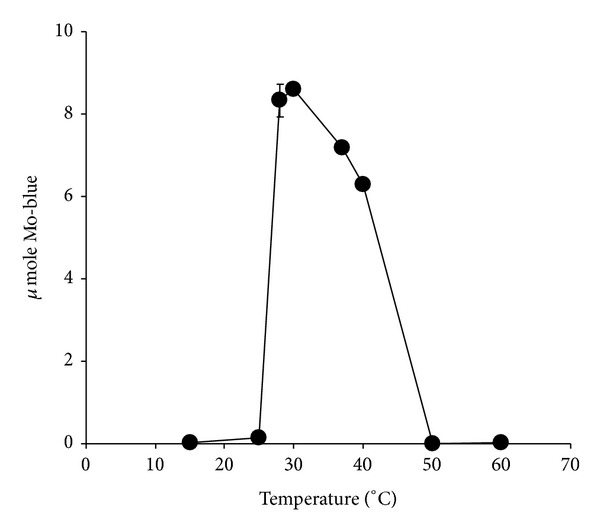
The effect of temperature on molybdate reduction by strain A.rzi. Error bars represent mean ± standard error (*n* = 3).

The optimum temperature supporting molybdate reduction was in the range from 28 to 30°C (Figure 5). The effect of pH was carried out using 50 mM of Tris and 10 mM of phosphate buffers spanning the pH from 7.0 to 9.0. The optimum pH for molybdenum reduction is pH 7.3 (data not shown). 

### 3.5. The Effect of Electron Donor Sources on Molybdate Reduction

**Figure 6:**
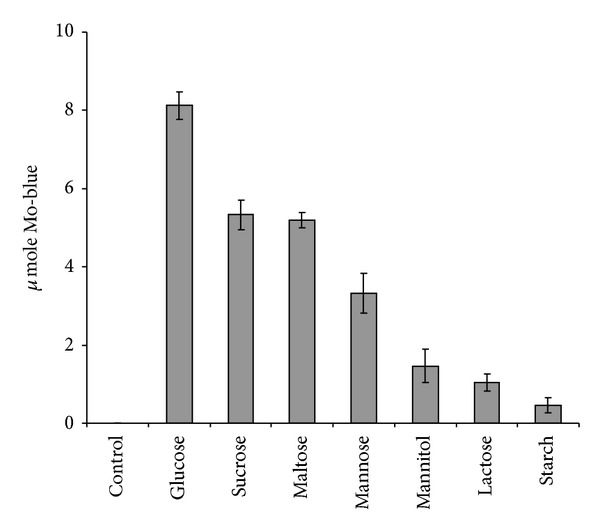
Molybdate reduction using various electron donor sources. Error bars represent mean ± standard error (*n* = 3).

Glucose was the most effective supplement as an electron donor for supporting molybdate reduction. This was followed by sucrose, maltose, mannose, mannitol, lactose, and starch (Figure 6). Glucose was optimum at 1% (w/v).

### 3.6. Mo-Blue Absorbance Spectrum

**Figure 7:**
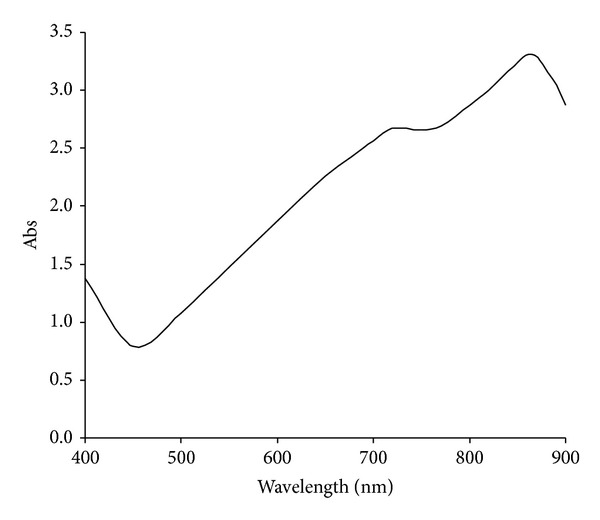
Scanning spectrum of Mo-blue from *Bacillus* sp. strain A.rzi after 24 hours of static incubation.

The spectrum of Mo-blue obtained from the growth medium shows a maximum peak near the far red region between 860 and 870 nm and a shoulder approximately at 710 nm (Figure 7). 

### 3.7. Studies on the Effects of Metal Ions and Respiratory Inhibitors

**Figure 8:**
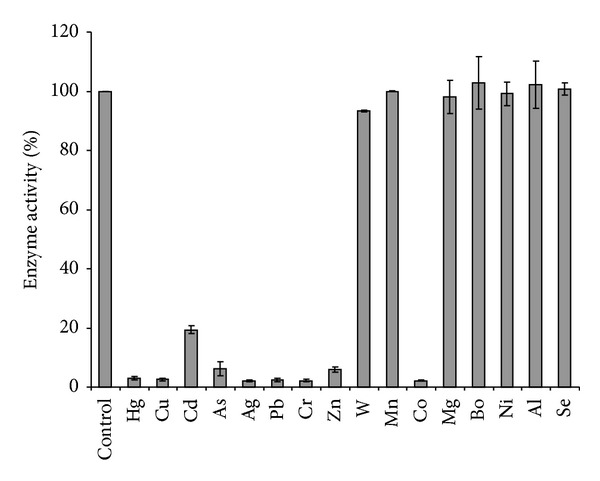
The effect of heavy metals (0.1 mM) on molybdate reduction by strain A.rzi. Error bars represent mean ± standard error (*n* = 3).

Preliminary results indicated that stannous and ferrous ions resulted in a chemical reduction of phosphomolybdate to Mo-blue in the reaction mixture. Hence these metal ions were omitted from this study. At 0.1 mM, mercury, copper, cadmium, arsenic, lead, chromium, cobalt, and zinc showed strong inhibition on molybdate reduction by crude enzyme (Figure 8).

It was found that the inhibitors antimycin A, potassium cyanide, sodium azide, and rotenone did not inhibit more than 10% of the Mo-reducing activity in strain A.rzi (data not shown). 

### 3.8. Characterization of the Molybdate Reduction Reaction Using the Dialysis Tubing Method

The results showed that 95% of the amount of Mo-blue formed (13.775 *μ*mole) was found in the dialysis tube, while only 5% (1.225 *μ*mole) was found at the outside of the tube.

### 3.9. Kinetics of Molybdenum Blue Production

**Figure 9:**
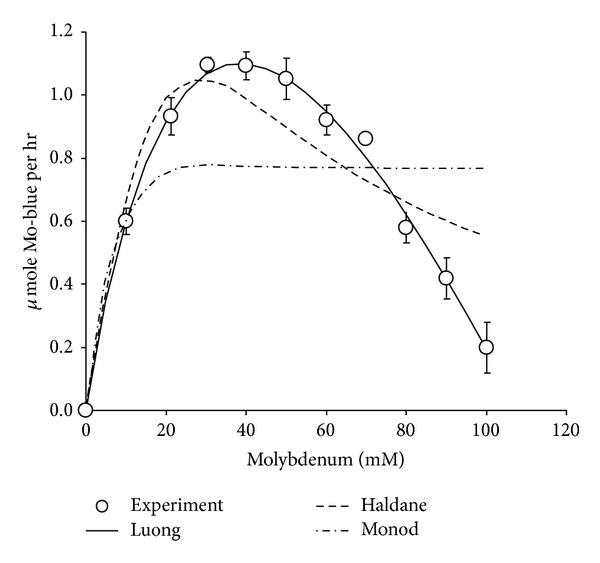
Kinetics of molybdenum blue production by strain A.rzi. Data represents mean ± standard error (*n* = 3).

Data from the experimental value in batch studies was fitted to several kinetic models of growth or product formation, that is, Monod, Luong, and Haldane. CurveExpert Professional software (Version 1.6) with custom equation algorithm that leads to the minimization of the sums of square of residuals was used to find the constants. The best model that fitted the experimental data well was Luong followed by Haldane and Monod with correlation coefficient values of 0.99, 0.83, and 0.36, respectively (Figure 9). The calculated value for *p*_max⁡_, *K*_*s*_, *S*_*m*_, and *n* was 5.88 *μ*mole Mo-blue hr^−1^, 70.36 mM, 108.22 mM, and 0.74, respectively. 

## 4. Discussions

In the last decade, works on microbial molybdenum reduction to Mo-blue have been restricted to our isolates [18–25, 35, 36, 42]. The enzyme responsible for the reduction has never been purified. Despite this, novel enzyme assay for this enzyme has been developed [35] and purification of this enzyme is being intensely pursued. Our quest for more variety of Mo-reducing bacteria to suit a plethora of environmental conditions has led us to the discovery of this bacterium. Molybdenum bioremediation can take advantage of this genus ability to produce spores that can be stored for long period and highly resistant to environmental stresses including heavy metals [43]. 

This bacterium was able to produce comparable molybdenum to other previously isolated strains. Furthermore it is able to reduce a high initial concentration of sodium molybdate of 80 mM. Average reduction at moderate starting molybdate concentrations from previous studies ranges from 25 to 55 mM [17–25]. Tolerance and reduction at concentrations higher than 20 mM are an advantage to a microbe as molybdenum pollution could reach as high as 2000 ppm (20.8 mM molybdate) [44], a concentration lethal to ruminant [45]. 

The optimum ratio of phosphate to molybdate concentrations is important for overall molybdenum reduction to molybdenum blue. The results obtained in this work show some similarity to the results obtained from *Serratia* sp. strain Dr.Y8 [19] and *S. marcescens *strain Dr.Y9 [21]. Other strains show a lower requirement for molybdenum of between 15 and 50 mM but with a similar optimal phosphate at 5 mM [18, 20, 22–25]. The inhibitory effect shown by high phosphate concentrations is probably due to physical interaction with the phosphomolybdate substrate and not through inhibiting the enzymatic action. This has been discussed in detail in other similar publications [18–25]. The Mo-blue spectrum obtained in this strain is similar to all of the other Mo-reducing strains [18–25, 42] indicating that a common phosphomolybdate species is involved.

Optimal pH and temperature supporting molybdenum reduction for this bacterium were found to be within the range of optimum pH and temperature of nearly all Mo-reducing bacteria isolated to date which varies from pH 6 to 8 and from 30 to 40°C, respectively [18–25]. The temperature range suits tropical climate bioremediation environment [46].

The discovery that glucose was the best electron donor to molybdenum reduction is similar to results obtained from several other Mo-reducing bacteria [20, 22, 24]. Support of Mo-reduction using starch is unique to this strain as this genus is known for its production of amylases [47]. Other Mo-reducing bacteria require sucrose [19, 21] or fructose [25]. Since molybdenum reduction is growth associated [18–25], the use of glucose and all of the other readily assimilable carbon sources reflect a growth-associated process of molybdenum reduction. This process is ubiquitous in this and other Mo-reducing bacteria. 

Toxic metal ions such as mercury, copper, cadmium, lead, chromium, and ions showed a similar trend of inhibition to other Mo-reducing bacteria [18–25]. The inhibition by arsenic, cobalt, and zinc is only reported for this strain. In a related area, the bioremediation of chromium is also affected by heavy metals ions [48–51]. 

The results obtained indicate that the electron transport chain (ETC) or system of this bacterium is not the site of molybdate reduction. The result is in agreement with many of the more recent isolated Mo-reducing bacteria [18–25]. In contrast, the electron transport chain has been suggested as the site of molybdate reduction in EC 48 [17]. The inhibitors inhibit at specific sites of the electron transport chain. Antimycin A is an inhibitor to cytochrome b. Rotenone inhibits NADH dehydrogenase. Sodium azide and cyanide are inhibitors to cytochrome d oxidase [37]. The same respiratory inhibitors have been used to find the location or identity of many metal-reducing enzymes with mixed results. It was discovered that rotenone, cyanide, and azide failed to inhibit chromate reduction in *E. coli* [50] and in *Pseudomonas mendocina* [51]. In contrast, both azide and cyanide were found to inhibit the reduction of chromate in *Bacillus subtilis* indicating the involvement of the ETC [52].

The dialysis tubing is the standard technique first developed by Hem [41] to distinguish between chemical and enzymatic reduction of metal ions by bacteria and has been modified to accommodate molybdenum reduction in bacteria [53]. The dialysis tubing experiment suggests that Mo-reduction is exclusively enzymatic in origin as the 5% of Mo-blue found at the outside of the tubing is almost all due to slow leakage of the Mo-blue formed. 

Most studies on the reduction kinetics of heavy metals such as mercury [54], arsenate [55], and chromate [56] reported a Haldane-type inhibition by the substrate metal ions. However, molybdenum reduction to Mo-blue showed a clear strong inhibition of Mo-blue production rate at high concentration of molybdenum with a calculated critical concentration of molybdenum that completely inhibited Mo-blue production at 108.22 mM. Unlike the more commonly reported Haldane model [39], the Luong model allows for the determination of the critical concentration of substrate that could completely inhibit production of product [40] as evident from this work. This is the first time existing models of kinetic studies being applied to model Mo-blue production in bacterium. 

To conclude, we reported on the isolation of a novel molybdenum reducing bacterium from the *Bacillus* genus. Features of this bacterium, such as temperature, pH, and concentration of phosphate that supported the optimal reduction of molybdenum blue, were similar to other reported molybdenum-reducing bacterial species. The absorption spectrum of the Mo-blue product was very similar to other Mo-reducing bacteria isolated to date indicating probably the same phosphomolybdate species involved in bacterial reduction process. The reduction of molybdenum to Mo-blue is predominantly enzymatic as evident from the dialysis tubing experiment. This bacterium is sensitive towards heavy metals as was similarly discovered in previously isolated molybdenum-reducing bacteria and could pose a problem if the bioremediation site is cocontaminated with other toxic heavy metals. We also showed for the first time that the Luong model of substrate (molybdate) inhibition kinetics of Mo-blue production was better than the Haldane model. We are currently focusing on the isolation of metal-resistant Mo-reducing bacteria and the purification of the Mo-reducing enzyme.

## Figures and Tables

**Table 1:** Various kinetic models for effect of substrate on Mo-blue production.

Author	Model	Author
Monod	$p=p_{\max}\frac{S}{K_{S}+S}$	Monod [38]
Haldane	$p=p_{\max}\frac{S}{S+K_{S}+\left(S^{2}/K_{i}\right)}$	Haldane [39]
Luong	$p=p_{\max}\frac{S}{K_{s}+S}{\left(\frac{1-S}{S_{m}}\right)}^{n}$	Mulchandani et al. [40]

**Table 2:** Amount of Mo-blue produced by a 24-hour static culture of strain A.rzi. Values are mean ± standard error (*n* = 3).

Bacteria	Micromole Mo-blue
*Bacillus *sp. strain A.rzi	7.82 ± 0.24^a^
*Serratia *sp. strain Dr.Y8	10.41 ± 0.13^b^
*S. marcescens *strain Dr.Y9	9.86 ± 0.44^b^
*Serratia *sp. strain Dr.Y5	7.87 ± 0.15^a^
*Pseudomonas *sp. strain DRY2	6.94 ± 0.65^c^
*Enterobacter *sp. strain Dr.Y13	6.91 ± 0.15^c^
*Acinetobacter calcoaceticus *strain Dr.Y12	5.86 ± 0.14^d^
*Serratia marcescens* strain DRY6	2.84 ± 0.23^e^
*Enterobacter cloacae* strain 48	2.17 ± 0.56^e^
*Escherichia coli* K12	0.96 ± 0.04^f^

Value with the same letter is not significantly different (*P* > 0.05).

## References

[1] Katnoria JK, Arora S, Bhardwaj R, Nagpal A (2011). Evaluation of genotoxic potential of industrial waste contaminated soil extracts of Amritsar, India. *Journal of Environmental Biology*.

[2] Shukor MY, Bakar NA, Othman AR, Yunus I, Shamaan NA, Syed MA (2009). Development of an inhibitive enzyme assay for copper. *Journal of Environmental Biology*.

[3] Alina M, Azrina A, Yunus ASM, Zakiuddin SM, Effendi HMI, Rizal RM (2012). Heavy metals (mercury, arsenic, cadmium, plumbum) in selected marine fish and shellfish along the straits of Malacca. *International Food Research Journal*.

[4] Davis GK, Merian E (1991). Molybdenum. *Metals and Their Compounds in the Environment: Occurrence, Analysis and Biological Relevance*.

[5] King RB, Long M, Sheldon JbK (1992). *Practical Environmental Bioremediation: The Field Guide*.

[6] DOE (2007). *Malaysia Environmental Quality Report 2006*.

[7] Fairhall LT, Dunn RC, Sharpless NE, Pritchard EA (1945). *The Toxicity of Molybdenum*.

[8] Underwood EJ (1979). Environmental sources of heavy metals and their toxicity to man and animals. *Progress in Water Technology*.

[9] Yong FS (2000). Mamut copper mine—the untold story. The national seminar on the Malaysian minerals industry. *Minerals: Underpinning Yesterday's Needs, Today's Development and Tomorrow's Growth*.

[10] Sayyed RZ, Chincholkar SB (2010). Growth and siderophores production in *Alcaligenes faecalis* is regulated by metal ions. *Indian Journal of Microbiology*.

[11] Capaldi A, Proskauer B (1896). Beiträge zur Kenntnis der Säurebildung bei Typhusbacillen und Bacterium coli. *Zeitschrift für Hygiene und Infectionskrankheiten*.

[12] Jan A (1939). La reduction biologique du molybdate d'ammonium par les bactéries du genre Serratia. *Bulletin des Sciences Pharmacologiques*.

[13] Woolfolk CA, Whiteley HR (1962). Reduction of inorganic compounds with molecular hydrogen by *Micrococcus lactilyticus*. I. Stoichiometry with compounds of arsenic, selenium, tellurium, transition and other elements. *Journal of Bacteriology*.

[14] Bautista EM, Alexander M (1972). Reduction of inorganic compounds by soil microorganisms. *Soil Science Society of America Journal*.

[15] Campbell AM, Campillo-Campbell AD, Villaret DB (1985). Molybdate reduction by *Escherichia coli* K-12 and its chl mutants. *Proceedings of the National Academy of Sciences of the United States of America*.

[16] Sugio T, Tsujita Y, Katagiri T, Inagaki K, Tano T (1988). Reduction of Mo^6+^ with elemental sulfur by *Thiobacillus ferrooxidans*. *Journal of Bacteriology*.

[17] Ghani B, Takai M, Hisham NZ (1993). Isolation and characterization of a Mo^6+^-reducing bacterium. *Applied and Environmental Microbiology*.

[18] Shukor MY, Habib SHM, Rahman MFA (2008). Hexavalent molybdenum reduction to molybdenum blue by *S. Marcescens* strain Dr. Y6. *Applied Biochemistry and Biotechnology*.

[19] Shukor MY, Rahman MF, Suhaili Z, Shamaan NA, Syed MA (2009). Bacterial reduction of hexavalent molybdenum to molybdenum blue. *World Journal of Microbiology and Biotechnology*.

[20] Shukor MY, Rahman MF, Shamaan NA, Syed MS (2009). Reduction of molybdate to molybdenum blue by *Enterobacter* sp. strain Dr.Y13. *Journal of Basic Microbiology*.

[21] Yunus SM, Hamim HM, Anas OM, Aripin SN, Arif SM (2009). Mo (VI) reduction to molybdenum blue by *Serratia marcescens* strain Dr. Y9. *Polish Journal of Microbiology*.

[22] Rahman MFA, Shukor MY, Suhaili Z, Mustafa S, Shamaan NA, Syed MA (2009). Reduction of Mo(VI) by the bacterium *Serratia* sp. strain DRY5. *Journal of Environmental Biology*.

[23] Shukor MY, Rahman MF, Suhaili Z, Shamaan NA, Syed MA (2010). Hexavalent molybdenum reduction to Mo-blue by *Acinetobacter calcoaceticus*. *Folia Microbiologica*.

[24] Shukor MY, Ahmad SA, Nadzir MMM, Abdullah MP, Shamaan NA, Syed MA (2010). Molybdate reduction by *Pseudomonas* sp. strain DRY2. *Journal of Applied Microbiology*.

[25] Lim HK, Syed MA, Shukor MY (2012). Reduction of molybdate to molybdenum blue by *Klebsiella* sp. strain hkeem. *Journal of Basic Microbiology*.

[26] Neunhäuserer C, Berreck M, Insam H (2001). Remediation of soils contaminated with molybdenum using soil amendments and phytoremediation. *Water, Air, and Soil Pollution*.

[27] Mohandass R, Rout P, Jiwal S, Sasikala C (2012). Biodegradation of benzo[a]pyrene by the mixed culture of *Bacillus cereus* and *Bacillus vireti* isolated from the petrochemical industry. *Journal of Environmental Biology*.

[28] Devereux R, Wilkinson SS, Akkermans ADL, van Elsas JD, de Bruijn FJ (2004). Amplification of ribosomal RNA sequences. *Molecular Microbial Ecology Manual*.

[29] Thompson JD, Higgins DG, Gibson TJ (1994). CLUSTAL W: improving the sensitivity of progressive multiple sequence alignment through sequence weighting, position-specific gap penalties and weight matrix choice. *Nucleic Acids Research*.

[30] Jukes TH, Cantor CR, Munro HN (1969). Evolution of protein molecules. *Mammalian Protein Metabolism*.

[31] Saitou N, Nei M (1987). The neighbor-joining method: a new method for reconstructing phylogenetic trees. *Molecular Biology and Evolution*.

[32] Felsenstein J (1985). Confidence limits on phylogenies: an approach using the bootstrap. *Evolution*.

[33] Margush T, McMorris FR (1981). Consensus n-trees. *Bulletin of Mathematical Biology*.

[34] Page RDM (1996). TreeView: an application to display phylogenetic trees on personal computers. *Computer Applications in the Biosciences*.

[35] Shukor MY, Rahman MF, Shamaan NA, Lee CH, Karim MIA, Syed MA (2008). An improved enzyme assay for molybdenum-reducing activity in bacteria. *Applied Biochemistry and Biotechnology*.

[36] Shukor MY, Shamaan NA, Syed MA, Lee CH, Karim MIA (2000). Characterization and quantification of molybdenum blue production in *Enterobacter cloacae* strain 48 using 12-molybdophosphate as the reference compound. *Asia Pacific Journal of Molecular Biology and Biotechnology*.

[37] Dawson RMC, Elliot DC, Elliot WH (1969). *Data for Biochemical Research*.

[38] Monod J (1949). The growth of bacterial cultures. *Annual Review of Microbiology*.

[39] Haldane JBS (1930). *Enzymes*.

[40] Mulchandani A, Luong JHT, Groom C (1989). Substrate inhibition kinetics for microbial growth and synthesis of poly-*β*-hydroxybutyric acid by *Alcaligenes eutrophus* ATCC 17697. *Applied Microbiology and Biotechnology*.

[41] Hem JD (1972). Chemical factors that influence the availability of iron and manganese in aqueous solution. *Geological Society of America Bulletin*.

[42] Shukor MY, Adam H, Ithnin K, Yunus I, Shamaan NA, Syed A (2007). Molybdate reduction to molybdenum blue in microbe proceeds via a phosphomolybdate intermediate. *Journal of Biological Sciences*.

[43] Runnells DD, Kaback DS, Thurman EM, Chappel WR, Peterson KK (1976). Geochemistry and sampling of molybdenum in sediments, soils, and plants in Colorado. *Molybdenum in the Environment*.

[44] Sinnakkannu S, Abdullah AR, Tahir NM, Abas MR (2004). Degradation of metsulfuron methyl in selected malaysian agricultural soils. *Fresenius Environmental Bulletin*.

[45] Hassan AM, Haroun BM, Amara AA, Ehab A (2013). Serour production and characterization of keratinolytic protease from new wool-degrading *Bacillus* species isolated from Egyptian ecosystem. *BioMed Research International*.

[46] Elangovan R, Philip L, Chandraraj K (2010). Hexavalent chromium reduction by free and immobilized cell-free extract of arthrobacter rhombi-RE. *Applied Biochemistry and Biotechnology*.

[47] Sau GB, Chatterjee S, Mukherjee SK (2010). Chromate reduction by cell-free extract of *Bacillus firmus* KUCr1. *Polish Journal of Microbiology*.

[48] Poopal AC, Laxman RS (2009). Studies on biological reduction of chromate by *Streptomyces griseus*. *Journal of Hazardous Materials*.

[49] Thacker U, Madamwar D (2005). Reduction of toxic chromium and partial localization of chromium reductase activity in bacterial isolate DM1. *World Journal of Microbiology and Biotechnology*.

[50] Shen H, Wang Y (1993). Characterization of enzymatic reduction of hexavalent chromium by *Escherichia coli* ATCC 33456. *Applied and Environmental Microbiology*.

[51] Rajwade JM, Salunke PB, Pknikar KM, Amils R, Ballester A (1999). Biochemical basis of chromate reduction by *Pseudomonas mendocina*. *Proceedings of the International Biohydrometallurgy Symposium*.

[52] Garbisu C, Alkorta I, Llama MJ, Serra JL (1998). Aerobic chromate reduction by *Bacillus subtilis*. *Biodegradation*.

[53] Shukor MY, Syed MA, Lee CH, Karim MIA, Shamaan NA (2002). A method to distinguish between chemical and enzymatic reduction of molybdenum in *Enterobacter cloacae* strain 48. *Malaysian Journal of Biochemistry*.

[54] Głuszcz P, Petera J, Ledakowicz S (2011). Mathematical modeling of the integrated process of mercury bioremediation in the industrial bioreactor. *Bioprocess and Biosystems Engineering*.

[55] Sukumar M (2010). Reduction of hexavalent chromium by *Rhizopus oryzae*. *African Journal of Environmental Science and Technology*.

[56] Soda SO, Yamamura S, Zhou H, Ike M, Fujita M (2006). Reduction kinetics of As (V) to As (III) by a dissimilatory arsenate-reducing bacterium, *Bacillus* sp. SF-1. *Biotechnology and Bioengineering*.

